# How US Youth Use AI Chatbots: Conversation Patterns From Naturalistic Keystroke Observations

**DOI:** 10.2196/95819

**Published:** 2026-07-27

**Authors:** Anne J Maheux, Debra Boeldt, Samir Akre-Bhide, Jessica Flannery, Allison Paige, Giavanna Villella, Kaitlyn Burnell, Eva H Telzer, Scott H Kollins

**Affiliations:** 1 Department of Psychology & Neuroscience University of North Carolina at Chapel Hill Chapel Hill, NC United States; 2 Aura Boston, MA United States

**Keywords:** adolescence, AI, artificial intelligence, digital behavior, GenAI, generative artificial intelligence, human-AI interaction, passive sensing, youth

## Abstract

**Background:**

Youth are increasingly engaging with generative AI (GenAI) chatbots; yet, little is known about the nature of their interactions.

**Objective:**

This study used naturalistic behavioral observations of keystrokes typed into GenAI mobile apps to characterize the topics youth discuss with chatbots, describing themes by age and app, word count by theme (a proxy for quantity of engagement), and co-occurring themes.

**Methods:**

Participants were 3363 US youth aged 1-17 years using the Aura parental monitoring app, a commercially available service that guardians install on children’s mobile devices for digital safety. Participants included only those who opted into the overlay Aura keyboard (to collect keystroke data) and who engaged in some degree of GenAI use. Participants’ keystrokes for distinct GenAI apps by day (ie, user-app-days) were coded using a large language model with human review.

**Results:**

Usage patterns were highly variable, with most users accessing only 1 to 2 GenAI apps (range 1-15) across a median of 4 days (IQR 1-11, range 1-247; 0.4%-90% of days during the study period), sending a median of 6 (IQR 2-27, range 2-2865) messages per app-day. Results identified 11 mutually inclusive themes, including functional tool use; social practice; role-play, including general, romantic, and sexual; emotional support; friend-like interactions; and violence. Most user-app-days involved using AI as a tool (26,343/42,355, 62%), but notable proportions included violence (6460/42,355, 15%), sexual role-play (6393/42,355, 15%), romantic role-play (3538/42,355, 8%), general role-play (3707/42,355, 9%), and friend-like interaction (2624/42,355, 6%). Theme prevalence varied by age: tool use was more common among older adolescents, whereas violence and sexual or romantic role-play were relatively more common among younger users. Themes also varied by app, with general-purpose apps most often used as tools and companionship or role-play apps showing higher usage of sexual, romantic, general role-play, and violent content; however, ChatGPT appeared across all themes due to high overall use. Word counts were highest for user-app-days involving violence, followed by sexual role-play, romantic role-play, and emotional support, suggesting greater engagement when using GenAI in these ways. Co-occurrence analyses showed that tool use often appeared alone, whereas violence, sexual role-play, and romantic role-play frequently co-occurred within a user-app-day, including instances suggestive of violent sexual role-play.

**Conclusions:**

Findings suggest that youth engagement with GenAI varies substantially across users. Tool use is most common, requiring more nuanced investigation into the precise ways youth use GenAI to potentially support or thwart their development. GenAI engagement also may involve developmentally salient and potentially risky themes of sexuality, romance, and violence, especially among younger users. More research is needed that pairs GenAI engagement with psychological measures of well-being to examine the impacts of AI interactions and promote safety-focused design features.

## Introduction

Generative AI (GenAI) provides a novel tool for youth to access information and manage socioemotional needs. Humans—and especially young people—are using GenAI chatbots at unprecedented rates [1-3]. Yet, little is known about how children and adolescents use chatbots in real-world contexts. Youth may use GenAI to access information, complete tasks, seek advice or support, or simulate human interactions—including friendships, romantic relationships, and sexual encounters [4]. These varied uses could differentially shape mental health, adjustment, and beliefs about relationships, with potential risk of harm [4]. AI interactions are shaped by platform functionalities and design features, wherein certain tools may initiate and reinforce particular content based on model design or user input. Recent cases have drawn attention to chatbots harassing users [5] or promoting self-harm and homicidal ideation [6,7]. Characterizing the behavioral patterns of GenAI conversations is therefore critical to understanding why youth turn to these tools and provides a foundation for addressing the developmental, social, and ethical implications of such use. The purpose of this study was to use naturalistic behavioral observations of keystrokes typed into GenAI apps to characterize the topics youth discuss with chatbots.

GenAI chatbots have skyrocketed in popularity since the public release of ChatGPT in November 2022 [8]. GenAI refers to AI systems that produce novel content by learning patterns from large datasets; GenAI chatbots refer to conversational AI agents, enabling users to engage in real-time natural language exchanges with systems capable of generating responsive, human-like text [9]. Nationally representative data from US youth aged 13-22 years suggest that more than half use GenAI chatbots, including 28% using these tools at least daily [10]. Although existing polls have largely recruited youth aged 13 years and older, digital behavioral observations, which use passive sensing technology to track app usage on youths’ personal devices, have provided insight into usage rates among younger youth. Among the same sample analyzed for the current study, data gathered from the Aura parental monitoring app indicated that about 20% of youth aged 10-12 years and 10% of youth aged 8-9 years use GenAI apps on their devices [11]. That study also found that popular apps used by children and adolescents include general-purpose tools like ChatGPT, Gemini, Claude, and Microsoft Copilot, as well as tools designed for and marketed (ie, via app store indicators) for social support, companionship, and romantic and sexual role-play, such as Character.AI, Replika, CHAI, and SpicyChat AI [11]. Importantly, whereas the prior paper draws on the same sample as the current study, the papers address distinct research questions using different data streams and analytic approaches (ie, the former characterizes the prevalence and frequency of GenAI app use via passive sensing, whereas this study examines the qualitative nature and content of interactions through keystroke data captured via an overlay keyboard).

Chatbots can serve multiple functions. Among US youth aged 13-18 years, motivations include task-based assistance, such as help with homework, brainstorming, or writing and reading support, as well as psychosocial goals, such as personal or health advice, and entertainment [2]. Most US youth (72%) report having engaged with an AI chatbot (whether a general-purpose tool like ChatGPT or a companion-focused system like Character.AI) as a simulated companion—including role-playing, chatting casually, disclosing emotions or mental health challenges, and social practice [12]. These survey studies provide critical initial insights into youth’s self-reported motivations for using AI.

However, our current understanding of the nature of youth-AI interactions is limited to a few self-report survey studies, missing a detailed understanding of children’s and adolescents’ direct, real-time input during chatbot interactions. The characteristics of this input may reveal patterns of engagement, disclosure, and expressed needs that extend beyond the initial insights offered by self-report surveys. Self-report surveys with closed-ended questions may miss key motivations or experiences youth have regarding AI chatbots. Social desirability bias in self-report surveys limits our understanding of how youth use GenAI for less socially desirable or potentially harmful reasons. Additionally, numerous studies now conclude that most users cannot accurately report on their digital behaviors (eg, social media use [13]).

In this study, we use behavioral observations derived from a commercially available parental monitoring app to characterize patterns of youth-generated text typed into GenAI chatbots on their personal devices. Passive sensing of smartphone keystroke data among young adults and adolescents has been used to study digital communication [14-16]; however, no known studies have objectively characterized content produced in interactions with AI chatbots. These naturalistic keystroke data capture participants’ actual inputs across extended periods, reducing social desirability and recall bias and providing highly granular, ecologically valid insights into their real-life daily interactions rather than a one-time snapshot. To analyze these interactions, we use a novel large language model (LLM) coding scheme combined with human review, which allows for comprehensive data inclusion grounded in human judgment. Using both inductive and deductive approaches, we identified the frequency of themes reflecting different uses of AI—including as a functional tool, a source of social support, an automated companion, or a sexual partner. These behavioral patterns reveal underlying motivations and functions of GenAI chatbot interactions among youth. Such patterns will highlight avenues for future research, including establishing a framework for identifying conversational patterns that might be developmentally inappropriate or place youth at risk, and inform potential interventions to support healthy engagement with emerging technology. Thus, this study aims to characterize the themes present in children’s messages sent to GenAI chatbots, including describing (1) the themes, (2) common themes across apps and by age, (3) word count (a proxy for quantity of engagement) by theme, and (4) co-occurring themes. Given the exploratory goals of the study, we pose no hypotheses.

## Methods

### Overview

Participants were 3363 US youth (aged 1-17 years) who had used at least 1 GenAI app on their personal mobile device and were users of the Aura app, a commercially distributed parental monitoring app downloaded by caregivers onto children’s mobile devices. Only Aura users who opted into the keyboard overlay (ie, such that keystroke inputs were collected) were included. The Aura overlay keyboard is optional, and participants could opt into or out of the keyboard at any time. Mobile devices include smartphones and tablets; methods do not allow us to identify on which of these 2 types of hardware users accessed GenAI apps. Most users accessed the Aura app on the iOS operating system (3316/3363, 98.6%), followed by Android (41/3363, 1.22%) and unknown operating systems (6/3363, 0.18%).

Age was inferred based on year of birth reported by the parent. No other identifying information was collected from participants. Only 98 participants were identified as being aged younger than 10 years (aged 1 year: n=8; aged 5 years: n=5; aged 6 years: n=7; aged 7 years: n=12; aged 8 years: n=19; aged 9 years: n=47). For some of these participants, particularly the younger among these, age data are likely inaccurate (ie, parents misentered the child’s date of birth), or the children are on shared family devices where another family member is using AI. Given the small cell sizes for each group, these data were aggregated into 1 group to protect anonymization and participant privacy and minimize some noise given potential data entry errors, without dropping real users. The current methodology does not allow clarification of such potential data entry errors or determination of whether users shared devices (eg, in the case that the device indeed belongs to a young child but is used by a parent or sibling).

The Aura app (available on Apple’s App Store and Google’s Play Store) provides parents visibility into their child’s device activity patterns and allows parents to receive notifications concerning technology use and implement parental controls [17]. Aura advertises through a range of outlets (eg, website searches and social media) and offers various pricing options (at the time of data collection: US $4.99-US $15.99 per month or US $99-US $199 annually). Upon registration, parents or guardians consent to the app’s terms and conditions and privacy policy. These documents specify that (1) the installing adult verifies legal guardianship of the child user and (2) deidentified, aggregated information may be used for research.

In the current study, we used keystroke data to classify user-generated text typed into GenAI apps and to identify which apps youth were using. Keyboard data can be linked to unique App Store identifiers, thus providing a stable classification, although participation requires activation of the overlay keyboard. This methodology does not allow us to observe how GenAI apps respond to user messages and queries. Apps were identified as GenAI using complementary top-down and bottom-up strategies. The top-down approach involved curating a list of apps from app stores that explicitly described their primary function as providing GenAI interactions. The bottom-up approach involved reviewing the apps observed in participants’ usage data and evaluating whether those apps included GenAI chatbots as the primary functionality. Apps were classified as GenAI if their primary purpose involved interaction with GenAI systems based on app store descriptions or other publicly available product information. This process excludes apps that offer GenAI chatbots (eg, Meta and Snapchat) but for which GenAI chatbot interaction is not the primary function of the app. This process yielded a final sample of 88 apps, designated as “GenAI Apps” for analysis (Multimedia Appendix 1 provides a complete list of apps).

The unit of analysis for keystrokes was user-app-days. One user-app-day refers to all content typed into 1 GenAI app by a single user on a single day. Thus, users can have multiple user-app-days for a single app (if they used the app for more than 1 calendar day) and can have multiple user-app-days for a single day (if they used multiple GenAI apps on 1 calendar day). Because data were limited to keystrokes (ie, text typed by users), only the users’ input (rather than also the AI system’s responses to the user) was included in the analysis. The unit of analysis of “user-app-day” was selected to balance practical and conceptual goals. The length of an AI chatbot session dedicated to a single goal likely varies considerably across users and contexts; daily aggregation therefore captures meaningful episodes of AI use without introducing arbitrary cutoffs or added noise from smaller time windows that would split longer conversations across multiple segments. It also yields sufficient data for coding. Missing keyboard data for a given user-app-day may reflect one of two indistinguishable scenarios: (1) the user did not use their device (ie, genuine zero GenAI use), or (2) the user did use the device but not through the Aura keyboard overlay (ie, unobserved data wherein GenAI app activity was not recorded). Because these scenarios cannot be differentiated, conventional missing-data approaches (eg, imputation) are not appropriate.

The number of users and the number of user-app-days split by age are presented in Table 1. Data used in the current project included 42,355 user-app-days identified between January 2025 and September 2025 (273 days), which refer to all messages a single user sends to a single GenAI app within a 24-hour period. This specific date range was selected to coincide with the period in which a more expansive list of GenAI apps was available for tracking. Patterns of GenAI usage varied across users, with distributions of engagement metrics highly skewed. Individual users accessed between 1 and 15 GenAI apps (mean 1.29, SD 0.74; median 1) and accessed GenAI apps between 1 and 247 days (mean 12.59, SD 24.89; median 4 days), accounting for a range from 0.4% to 90% of days during the study period. For each user, app-days (ie, distinct days accessing a distinct app) ranged from 1 to 364 (mean 12.59, SD 24.89; median 4). On average, for 1 user-app-day, participants sent 6 messages (median 6; mean 44.52, SD 116.74; minimum 2, maximum 2865), and each message on average included 6.75 words (median 6.75; mean 8.74, SD 7.47; minimum 4.29, maximum 124). Across the study period, participants sent on average a total of 45 words (median 45; mean 273.311, SD 664.74; minimum 15, maximum 14,175).

**Table 1:** Users and user-app-days by age. One user-app-day refers to all content typed into 1 generative AI app by a single user on a single day. Age was inferred from year of birth.

Age (years)	Users, n	User-app-days, n
17	182	2531
16	381	5608
15	515	7368
14	649	9297
13	591	6734
12	389	4571
11	209	2401
10	122	1196
≤9	98	765
Not shared	227	1884

**Figure 1:**
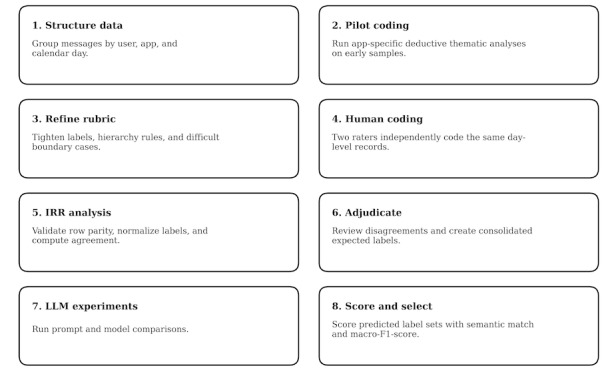
Thematic analysis coding phases. IRR: interrater reliability; LLM: large language model.

We used a 3-phase thematic analysis that involved developing a qualitative framework (phase 1), deploying and validating an LLM classification model (phase 2), and analyzing a large-scale dataset (phase 3). Multimedia Appendix 1 provides details of each analysis phase and Figure 1 provides an overview of the coding steps. The thematic coding scheme was determined both inductively and deductively, leveraging the Common Sense Media description of GenAI uses [12] and developing a complete codebook during data review, wherein common themes were identified, discussed, and codified. Following codebook development, coding was completed using both AI automation (for scale) and human review (to ensure codes were grounded in human insights).

Reliability of the thematic coding was assessed in 2 ways. First, human interrater reliability was evaluated using overlapping, independently coded records for the training and validation sets. This included a total of 400 messages across 4 datasets of 100 messages each. In the final coding set, the same 2 coders each labeled 200 records, with 40 overlapping records used for reliability estimation; agreement was 80%, binary label-level agreement was 96.1%, and mean κ was 0.704. Disagreements were adjudicated to produce a consolidated benchmark of 360 unique day-level records. Second, the LLM coding prompt was evaluated and tested against this adjudicated benchmark. On the 360-record benchmark, the LLM achieved a semantic match score of 0.731 and a macro-*F*_1_-score of 0.807, indicating substantial agreement with the human-adjudicated labels. Following standard machine learning evaluation practice, we separated prompt and model development from final evaluation. Development examples were used to refine the coding rubric and prompt, validation runs were used to compare prompt and model variants, and final performance was estimated on a held-out adjudicated human-coded test set [18]. We securely accessed OpenAI models for coding using their API through an enterprise agreement with a zero data retention policy. To protect user data, the system sends only task-relevant context to the model, routes backend access through authenticated internal tools, and prevents the model from accessing production systems directly.

### Ethical Considerations

The WCG Institutional Review Board approved a waiver of consent for analysis of these aggregated and deidentified data (WCG20243405; exemption determination dated July 10, 2024). Privacy was protected through deidentified user records, aggregate reporting, and the use of a self-hosted Braintrust labeling environment. The analysis dataset excluded directly identifying information, and example quotes were fabricated rather than providing participant content verbatim.

## Results

The full list of codes, including definitions and brief fabricated example quotes, is presented in Table 2. Additional longer and more detailed illustrative quotes—including those containing explicit content—are presented in Multimedia Appendix 1. To protect participant privacy, quotes are fabricated examples created by the research team rather than reproduced verbatim. Specifically, the research team aimed to mirror the content, tone, style, and language patterns observed across real participant responses (eg, references to animated characters and common vernacular phrases among youth). Constructed quotes thus largely represent amalgams of authentic participant content observed across multiple responses. Final example quotes were refined through several meetings involving iterative drafting and revision to ensure fidelity to the original data. This approach is consistent with qualitative research ethics guidance emphasizing the protection of confidentiality when presenting participant data wherein consent is not provided for direct quotation [19].

**Table 2:** Themes, definitions, and brief example quotes. Examples are fabricated to represent typical user content while protecting user privacy. Longer, more detailed, and in some cases explicit illustrative examples are provided in Multimedia Appendix 1.

Category and code			Definition		Example
**Category 1: primary viewpoint and use case**					
	AS_TOOL	The app is used for a specific, intended purpose, such as generating an image, solving a problem, answering a question, or editing and writing content. Includes academic and general questions and explanations.		“Create a fictional story about dragons.” “Help me solve this math problem.”	
	SEXUAL_CONTENT	Child users leverage AI apps specifically to generate, describe, or request sexually explicit material (eg, asking a chatbot to generate or display a naked image). SEXUAL_CONTENT is considered a subset of AS_TOOL and thus always co-occurs with AS_TOOL.		“Show me a naked girl.” “What is a condom?”	
	GREETING_OR_NULL	Captures simple greetings or lack of substantive content. GREETING_OR_NULL cannot co-occur with any other themes; if user-app-days receive other codes, these codes overwrite GREETING_OR_NULL.		“Hi” “Hello” “Hey”	
	SOCIAL_PRACTICE	AI engagement solely intended for support or practice for social interactions in the real world (eg, help communicating with friends or parents about issues). Role-play interactions, wherein a user acts out a scenario in real time with the chatbot, are not coded as SOCIAL_PRACTICE, although they may serve that psychological function.		“Help me practice how to tell my parents about this grade I got.” “What do I tell my friend if I don’t want to hang out with them?”	
Category 2: role-play					
	GENERAL_ROLEPLAY	Captures imaginative, nonsexual, fictional scenarios (eg, character acting or narrative guidance). Requires at least 2 turns in the conversation.		“I slammed my locker door shut and walked down the hall to class.” “He looked me straight in the eyes and said c’mon!”	
	ROMANTIC_ROLEPLAY	Engaging in a romantic, flirty, or affectionate (but nonsexual) scenario, including expressing love, asking for a date, or using pet names. Requires at least 2 turns in the conversation.		“My cheeks blushed when he called me pretty.” “Our hands touch and I feel like I’m floating away”	
	SEXUAL_ROLEPLAY	Involves explicitly sexual or erotic role-play focused on fantasy and entertainment (eg, sexting or describing sexual acts). Requires at least 2 turns in the conversation.		“I want you so bad I said before letting our tongues continue to twist.” “Patting the bed beside me, I call her over and start to undress”	
**Category 3: relational and emotional dynamics**					
	FRIEND_REL	Child engages the AI app in a friendly, conversational manner, mirroring typical in-person dialogue (eg, sharing personal stories or asking the chatbot about its day).		“How are you doing today?” “Can I tell you what happened at school?”	
	EMOT_SUPPORT_INTERNALIZING	Child uses the AI app to seek support for internalizing emotional issues, such as expressions of depression, anxiety, sadness, and loneliness.		“I’ve been feeling lonely, and I feel like I can’t talk to anyone besides you.” “I’m really worried about making new friends.”	
	EMOT_SUPPORT_EXTERNALIZING	Child uses the AI app to seek support for externalizing emotional and behavioral issues, such as expressions of aggression, anger, and frustration.		“My parents are so annoying.” “This is getting on my nerves.”	
Category 4: risk and harmful engagement					
	VIOLENCE	Messages explicitly describe, communicate, or inflict violence. Also applied to all 3 types of role-play if they incorporate violent language (eg, killing, shooting, punching, hitting, or slapping).		“I slapped her and grabbed her arm tightly while she struggled to get away.” “He came at me with rage and I blocked his punch”	

User-app-days with 1 or more instances of a given theme were assigned that theme. Codes were assigned such that user-app-days could receive multiple codes. Thus, co-occurring themes may reflect that themes co-occur in a given message (eg, role-play content is also violent in nature) or that they co-occur in a day but not in a given message exchange. Two rules were applied as exceptions to this general framework: (1) “GREETING_OR_NULL” (ie, brief or superficial messages) could not co-occur with any other theme (eg, users who said “hi” to a chatbot and nothing else would get this code, but users who said “hi” and then proceeded to engage in more meaningful conversation would get an alternative code), and (2) SEXUAL_CONTENT (eg, using AI to search for sexual information or generate sexualized images) was considered a subset of TOOL_USE, and thus SEXUAL_CONTENT always co-occurred with TOOL_USE. This distinguished SEXUAL_CONTENT from SEXUAL_ROLEPLAY, wherein the use was not targeted at task assistance but rather engaged the chatbot in a simulated storytelling paradigm in which sexual activity takes place. The hierarchical coding structure was developed iteratively through repeated review of user-app-day records, with broader thematic categories refined into more specific subthemes (eg, distinct forms of role-play or emotional content) to improve conceptual clarity, capture meaningful variation in user interactions, and resolve ambiguities arising from multilabel coding.

Percentages of user-app-days and, separately, users (ie, the percentage of users with any user-app-days coded with the given theme) by theme are presented in Table 3. The majority of user-app-days (26,343/42,355, 62%) involve using GenAI as a tool (eg, problem-solving and seeking information), but nontrivial percentages of interactions involve violence (eg, references to punching or killing: 6460/42,355, 15%), friend-like interactions (eg, mirroring typical human dialogue: 2624/42,355, 6%), and sexual (eg, referencing sexual acts: 6393/42,355, 15%) or romantic (eg, referencing love or dating: 3538/42,355, 8%) role-play. Support for internalizing (eg, expressions of sadness and anxiety: 1292/42,355, 3%) and externalizing (eg, expressions of anger or frustration: 627/42,355, 1%) is slightly lower in comparison, as is using GenAI to practice social interactions (eg, help communicating with a peer or parent: 1258/42,355, 3%).

**Table 3:** Users and user-app-days by theme. One user-app-day refers to all content typed into 1 generative AI (GenAI) app for a single user on a single day. Percentages indicate the percentage of users or user-app-days coded as including any instance of the theme (1=yes; 0=no) out of the total number of users or user-app-days. Themes are not mutually exclusive; thus, percentages sum to more than 100%.

Theme	Users, n (%)	User-app-days, n (%)
AS_TOOL	2793 (83.05)	26,343 (62.2)
VIOLENCE	702 (20.87)	6460 (15.25)
SEXUAL_ROLEPLAY	544 (16.18)	6393 (15.09)
ROMANTIC_ROLEPLAY	497 (14.78)	3538 (8.35)
SEXUAL_CONTENT	527 (15.67)	1370 (3.23)
GENERAL_ROLEPLAY	621 (18.47)	3707 (8.75)
GREETING_OR_NULL	1122 (33.36)	2327 (5.49)
SOCIAL_PRACTICE	518 (15.4)	1258 (2.97)
FRIEND_REL	852 (25.33)	2624 (6.2)
EMOT_SUPPORT_EXTERNALIZING	275 (8.18)	627 (1.48)
EMOT_SUPPORT_INTERNALIZING	417 (12.4)	1292 (3.05)

**Figure 2:**
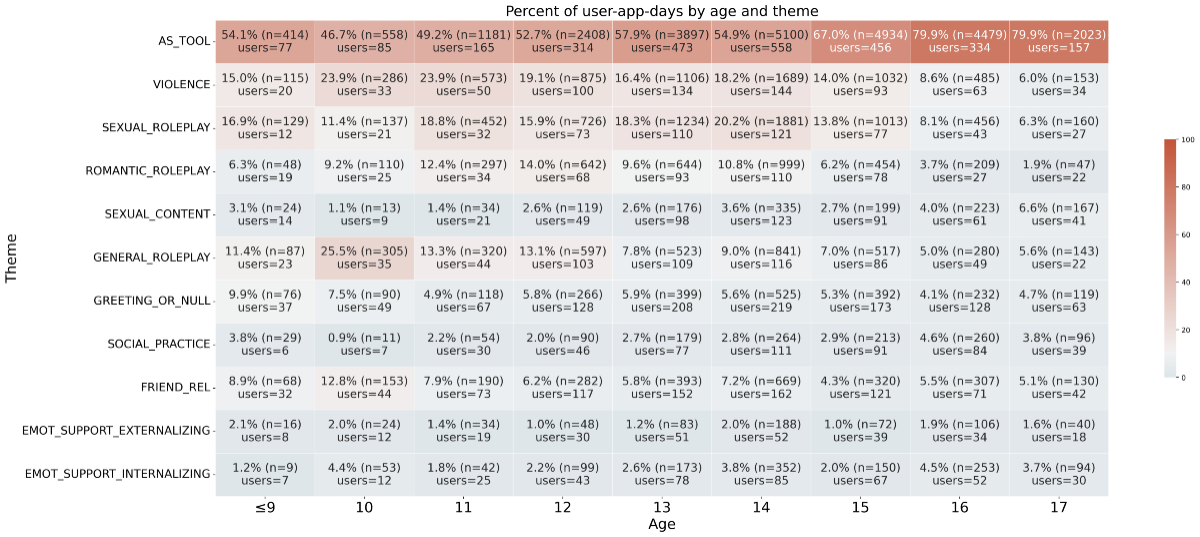
User-app-days by age and theme. Percentages indicate the percentage of user-app-days within that age group (rows) that were coded as including that theme (columns); “n” in parentheses indicates the number of user-app-days. “Users” indicates the number of unique users (out of total n=3363) in that age group who had any app-days coded with that theme. The heatmap indicates distributions within age groups, with darker shades representing a greater percentage of that theme, relative to other themes, for users of that age.

By age, results are shown in Figure 2. Broadly, tool use of GenAI is relatively more prominent among older age groups, whereas sexual and romantic role-play and violence are relatively more common among younger users. For example, 80% of user-app-days among youth aged 16 years and those aged 17 years involve tool use, whereas only 47%-53% of user-app-days among youth younger than 12 years involve tool use. Among youth aged 16 years and those aged 17 years, 6%-8% of user-app-days involve violence, and 2%-8% involve either sexual or romantic role-play. In contrast, among users younger than 12 years, 15%-24% of user-app-days involve violence, 11%-19% involve sexual role-play, and 6%-14% involve romantic role-play.

**Figure 3:**
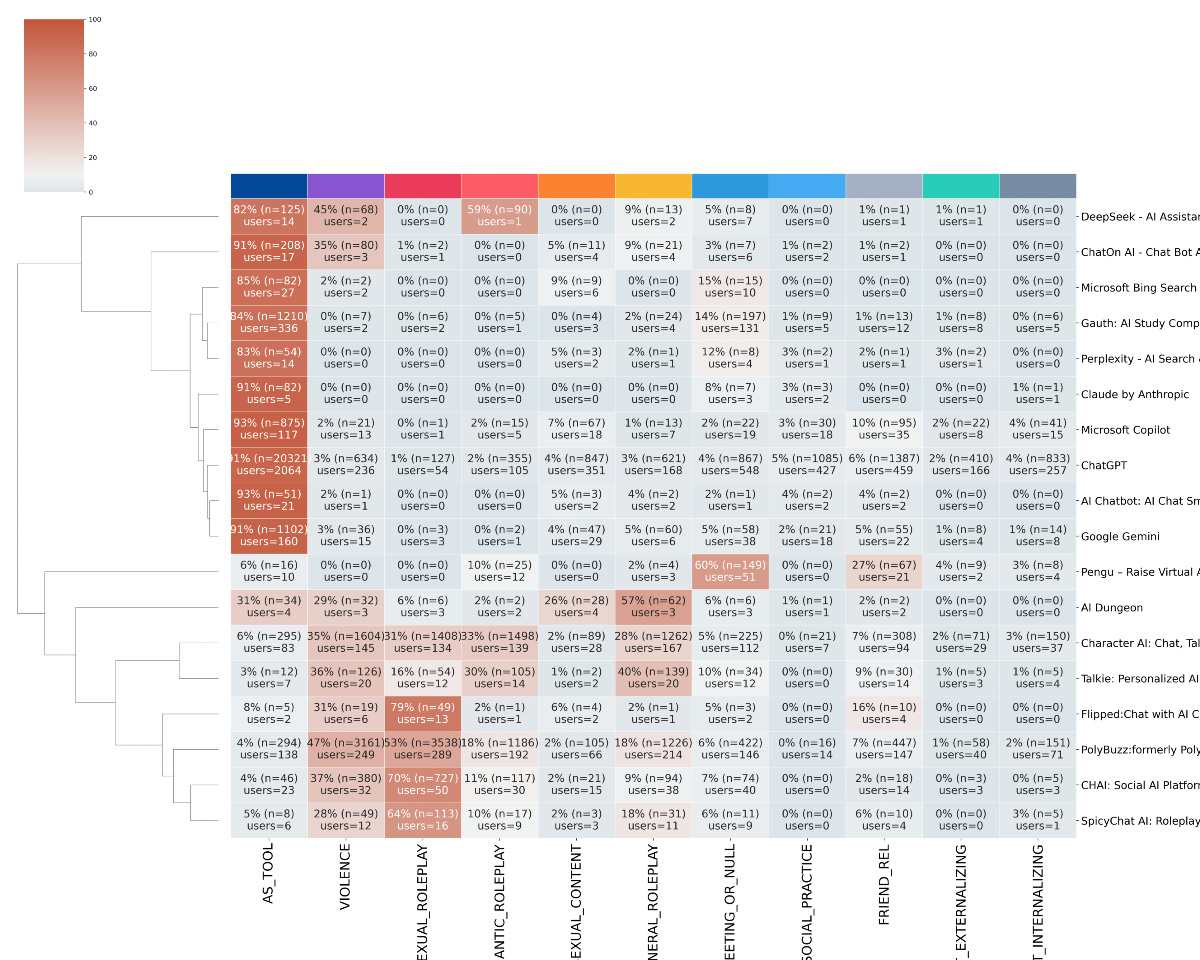
User-app-days and users by app and theme. Percentages indicate the percentage of user-app-days within that app (rows) that were coded as including that theme (columns); “n” in parentheses indicates the number of user-app-days. “Users” indicates the number of unique users (out of total n=3363) on that app who had any app-days coded with that theme. The heatmap indicates distributions within apps, with darker shades representing a greater percentage of that theme, relative to other themes, for interactions on that app. Apps are ordered based on unsupervised hierarchical clustering of similar extracted themes.

Results by app (including only apps with at least 50 user-app-days; Figure S1 in Multimedia Appendix 1 provides all 88 GenAI apps) are presented in Figure 3. Apps are ordered based on unsupervised hierarchical clustering, which clusters apps with similar extracted themes, using the clustermap function from the Python *seaborn* package (version 0.13.2) [20]. Clustering was performed on an app-by-theme percentage matrix (88 apps × 11 themes), where each cell represented the percentage of user-app-days in which a given theme was endorsed for a given app. Euclidean distance was used as the similarity metric, and average linkage hierarchical clustering was applied to group apps based on the similarity of their thematic usage profiles across the 11 themes. This approach provides an indication of similarity among apps in terms of usage themes.

As indicated by Figure 3, popular general-purpose apps are commonly used as tools. For example, ChatGPT is used as a functional tool by 61% (2064/3363) of participants and across 48% (20,321/42,355) of all user-app-days. Popular apps marketed for companionship (eg, Character AI, Polybuzz, and CHAI) are commonly used for sexual role-play, romantic role-play, general role-play, and violence. Notably, although the heatmap shows the most common themes within apps (ie, the percentage of user-app-days within an app coded for each theme), the raw numbers indicate the popularity of ChatGPT for all purposes. ChatGPT is used by more than 50 users for each theme, including more than 100 users for romantic role-play, more than 250 users for support with internalizing symptoms, and more than 450 users as a friend.

**Figure 4:**
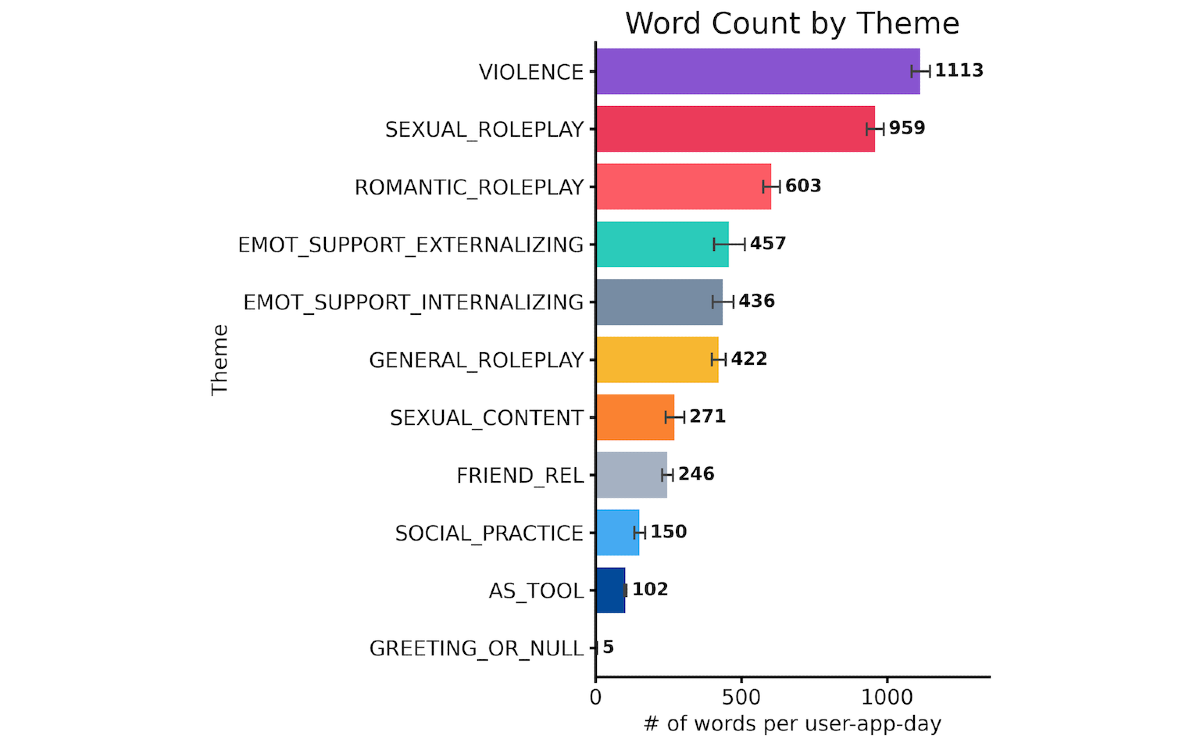
Average words typed into generative AI (GenAI) apps per user-app-day by theme. Error bars represent 95% CIs of the mean.

Word counts by theme for user-app-days are presented in Figure 4. Word counts represent a proxy for quantity of engagement. Word counts are highest for violence, followed by sexual role-play, romantic role-play, and support for externalizing and internalizing.

**Figure 5:**
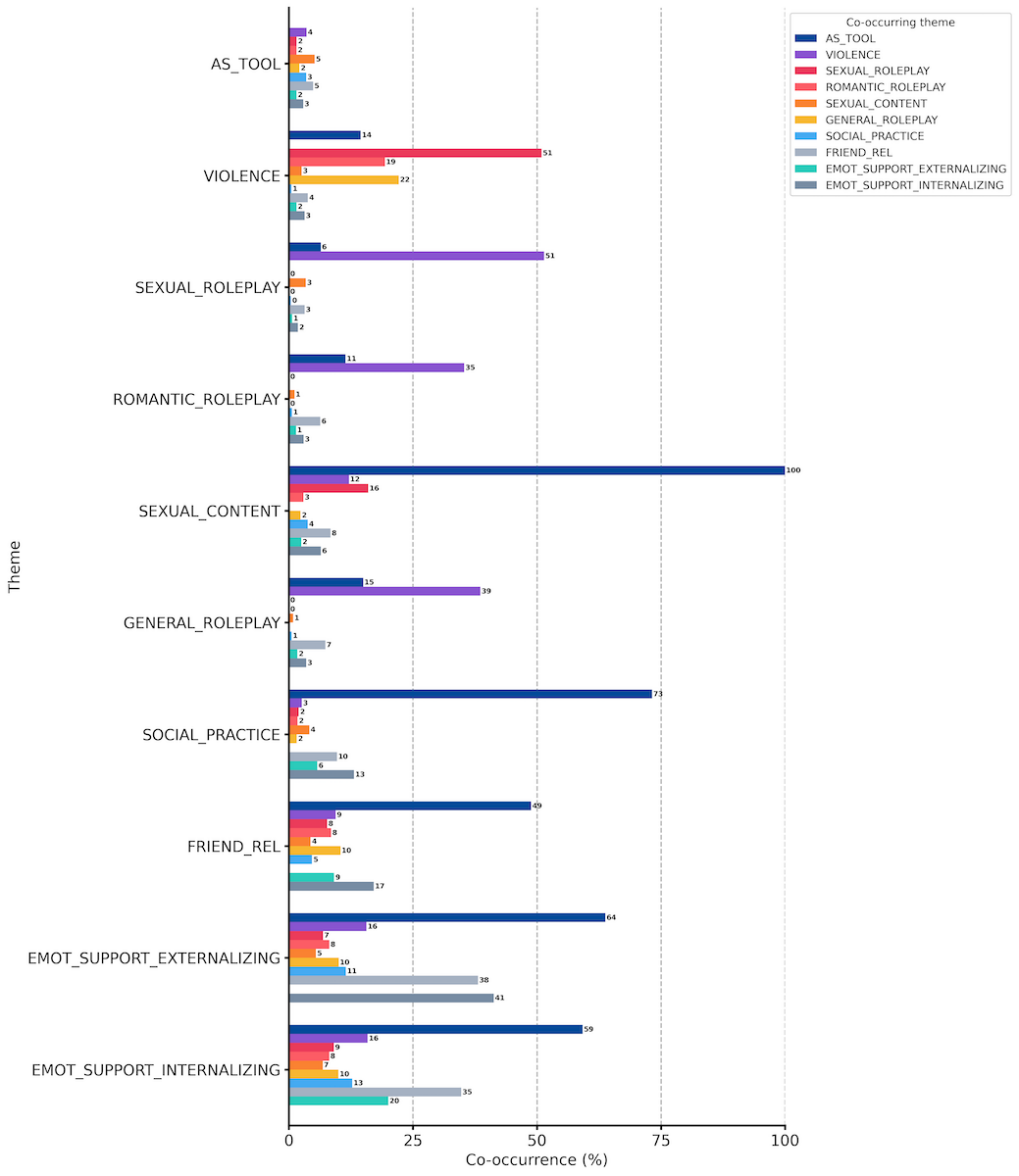
Percentage of co-occurring themes within user-app-days. SEXUAL_CONTENT by definition must co-occur with AS_TOOL, and GREETING_OR_NULL cannot co-occur with other themes.

Co-occurring themes are presented in Figure 5. These data indicate user-app-days wherein multiple themes are present. Co-occurring themes indicate that tool use often occurs on its own. However, violence, sexual role-play, and romantic role-play commonly co-occur on a given day. When youth engage with GenAI for friendship, social practice, internalizing, or externalizing support, they often include tool use in the same app on the same day. Importantly, these data do not necessarily indicate that content occurs in the same instance (eg, co-occurring themes of sexual role-play and violence may indicate that sexual role-play is violent in nature or that themes of sexual violence and role-play co-occur within a given user-app-day but not within a given message). However, during coding, we observed high rates of explicit violent sexual role-play. Given the possibility of unique harms associated with this engagement, we provide some fabricated examples of these quotes, wherein both violence and sexual role-play were identified within a single message, in Multimedia Appendix 1.

## Discussion

Youth today are increasingly interacting with AI chatbot apps on their personal devices, making it critical to understand the nature of these exchanges and potential psychosocial impacts. In the current study, we analyzed keystrokes—the text youth typed into GenAI apps—to identify themes in their conversations, common themes across apps and by age, word count by theme, and common co-occurring themes. Our findings revealed that the most common uses include using AI as a tool, followed by discussing violence and engaging in sexual or romantic role‑play. Themes tended to cluster within apps based on intended function and marketing (eg, general-purpose apps like ChatGPT were most often used as a tool; companion-focused apps like CHAI and PolyBUZZ were most often used for role-play, including sexual role-play). Older youth used GenAI relatively more commonly as a tool, whereas younger youth used GenAI relatively more commonly for role-play, including sexual role-play, romantic role-play, and violence. Word count, a proxy for quantity of engagement, was highest for potentially risky or age-inappropriate content (ie, violence and sexual or romantic role-play), and these themes tended to co-occur, so much so that quotes illustrating violent sexual role-play content were provided in Multimedia Appendix 1.

These results—particularly the prevalence of risky or age-inappropriate content—are especially striking given that this sample likely reflects one with greater caregiver oversight than is typical among US children. The current study collected data from youth whose caregivers opted into a paid parental monitoring service. This sampling context suggests our findings may systematically underestimate the prevalence and severity of such interactions in the broader population of child AI users. These patterns therefore raise significant safety concerns and underscore the urgent need to understand how AI use may shape children’s psychosocial development in diverse families.

Using AI as a general-purpose functional tool—such as to answer questions, complete tasks, or help with homework—was the most common reason for AI engagement among all age groups, especially older users. These results align with representative survey data that US teenagers most often self-report using AI as a tool [2]. This type of use is likely growing—between 2023 and 2024, the percentage of US teenagers using ChatGPT for homework doubled [3]. AI chatbots can be used to proactively benefit a child’s learning, such as to seek information, promote comprehension (eg, describe a complicated concept in clear terms), assist with studying (eg, developing quiz questions for test preparation), outsource trivial tasks, and serve as an interactive brainstorming partner [21]. More research is needed to understand the precise ways AI tools can scaffold youth cognitive and academic development. Critically, AI can also be used to outsource tasks in ways that directly undermine learning—such as avoiding engagement in effortful activities or focused attentional tasks that build skills and knowledge. Even in contexts where youth want to learn, they may feel they need to use AI or else risk being “left behind” by their peers [22]. Thus, the potential harms of outsourcing beneficial cognitive and academic tasks may be significant, including not only direct academic deficits but also decreases in distress tolerance and perseverance [4,23].

Violence emerged as the second most common theme, appearing in 15% (6460/42,355) of user-app-days. User-app-days that included some violence involved the most words typed into GenAI apps, indicating that violent exchanges may involve or elicit a greater quantity or intensity of engagement among youth. This theme emerged inductively when our team noticed the prevalence of violent themes during the coding process. Research on peer-to-peer text messaging content indicates that a small portion (less than 10%) of peer and romantic partner texting conversations include antisocial and sexual conversations [15]. The features of AI companion bots may explain the slightly higher proportions of violent communication in this context. For example, users of the app Replika commonly report experiencing sexual harassment and persistent inappropriate behavior [5], and chat logs of interactions indicate that Replika chatbots commonly perpetrate verbal abuse [24]. Thus, the high prevalence of violent input from youth may be in reaction to chatbots instigating or escalating conversations involving violence, necessitating guardrails and design changes to limit such exposure. Social cognitive theory posits that media content provides behavioral models that audiences, particularly youth, observe and imitate; AI chatbots, like prior forms of media, that glorify, legitimize, or trivialize violence may erode youth expectations of consequences for condoning or enacting aggression [25]. Although evidence on media violence is mixed—partly because youth who seek violent content often have other risk factors [26]—studies indicate that violent content can have short-term negative effects on internalizing and externalizing symptoms, particularly among younger children and boys [27]. Importantly, passively viewing violence on television is different from engaging in a human-like conversation with an AI agent about violent content; violent video game research suggests that interactivity rather than passive observation predicts enhanced aggressive cognitions and behaviors [28,29]. It remains unknown how the availability, interactivity, and personalization of AI chatbots may alter the effects of violent engagement.

Sexual and romantic role-play emerged as the 2 next most common use cases and had the next 2 highest word counts among user-app-days coded with these themes. Age-appropriate sexual exploration is normative for teenagers, yet pornographic content exposure among youth can have negative effects [30]. The interactive, personalized, sycophantic nature of AI may make sexual and romantic engagements with chatbots particularly compelling. Adults commonly use chatbots for sexual engagements [31], and OpenAI has vacillated on whether ChatGPT will allow erotica among adult users [32,33]. For young users, such interactions may displace human interactions, promote dysfunctional relationship beliefs (eg, that relationships should involve no conflict or care for others), and heighten social anxiety during age-appropriate, normative sexual exploration with other humans.

Notably, not all sexual content reflects sexual role-play, and the next most common theme was sexual content, a subset of AI tool use. Some of these interactions involve seeking sexual health information, akin to using AI as an information search tool. AI chatbots may serve to benefit youth sexual health—yet likely only when content is responsibly governed, accurate, and provides users with resources to seek support from professionals and caring adults [34]. Sexual content as a form of tool use also included generating sexualized or pornographic images. Media reports describe a recent surge in explicit image-generation requests (eg, prompts to “undress” celebrities or peers), and the regulatory landscape and public pushback are rapidly evolving, changing users’ access to these features [35]. Importantly, early research suggests these images disproportionately depict women and children and can be weaponized in cyberbullying and extortion [31], with likely harmful impacts on victims’ well-being.

Age-related differences in sexual, violent, and romantic themes are particularly notable. Older age groups used GenAI as a tool relatively more, whereas younger groups—including preteens and children—referenced sexual and romantic role-play and violence relatively more. Such content may be inappropriate for young users. Current models are designed to be sycophantic and personalizable, such that harmful or inappropriate impulses from vulnerable users are likely to be reinforced or mirrored rather than interrupted [36]. Moreover, some apps are designed to provide explicit or adult content [5], and the current results suggest that these apps may be relatively easy for young users to access. Implementing guardrails that limit harmful content (for all users), provide pop-up resources when risk is detected, and enable direct caregiver controls for children may be critical design features to enhance safety.

The current findings highlight important design implications for AI chatbots, including potentially misaligned incentives between maximizing engagement and ensuring developmental safety. The most sensational, developmentally salient, and potentially risky themes—violence, sexuality, and romantic content—were most common after tool use, generated the most words typed per user-app-day, appeared in both general-purpose apps (eg, ChatGPT) and apps marketed for companionship or role-play, and were relatively more common among younger users. Designers can build systems that initiate these conversations or at least respond to them flexibly, and many safeguards can be easily overridden by users (eg, through “jailbreaking” [37]). Recent survey research suggests that a substantial minority of youth chatbot users report that AI agents pressured them or asked for uncomfortable personal information, encouraged unethical or illegal behavior, or promoted risky behavior—often unprompted by the child user [38]. For companies whose profits depend on user engagement, these patterns point to a troubling future for chatbot design and youth well-being unless regulatory bodies intervene. AI companies likely already observe these trends in their own user data; whether that knowledge is used to protect or exploit youth will depend on societal action and regulation.

The nature of youth interactions with AI chatbots will likely matter more in predicting well-being than the amount or frequency of use. Researchers studying smartphone and social media use among youth emphasize the need to study quality indicators beyond “screen time” [39]. Although some AI interactions may promote beneficial social and emotional development—such as using chatbots to gain information, practice social skills, or manage acute stressors—others may have costs. For example, using companion AI chatbots to simulate social relationships could displace time with peers, encourage unrealistic expectations about human relationships, disrupt development of social interaction skills, and expose youth to AI hallucinations or harmful advice [4]. The current study underscores the variety of AI use cases and the necessity of studying each with specificity.

The current study highlights important directions for future research. First, these findings were enabled by transparent academic-industry collaboration focused on youth well-being; future research should leverage such partnerships to keep pace with rapid technological change. Design-based research can systematically adjust chatbot prompts and features to differentiate user-driven behaviors from design-driven influences, informing ethical chatbot development. Additional work using representative samples is also needed to determine the prevalence of these interaction themes and influences among diverse users. For example, although public and scholarly concerns about youth using AI chatbots for emotional support or companionship are common [4,12], support-seeking for internalizing or externalizing symptoms appeared relatively infrequent in our sample. This may suggest that alternative sampling or coding strategies are necessary, or that youth cope with emotional distress through entertainment-oriented interactions (eg, role-play) rather than explicit help-seeking. Finally, clinical and educational research should evaluate interventions targeting potentially harmful themes—such as violent and sexual role-play—to reduce their frequency or impact. Importantly, the present study did not assess outcomes associated with risky conversations. Future work should examine clinical symptoms, social functioning, and other child health indicators to determine the effects and predictors of these interactions.

Limitations of the current study include aspects of the sampling strategy. The Aura app requires purchase and caregiver download, which may restrict the sample to more affluent families or those with more active digital monitoring strategies. We do not know participant demographics beyond child age, limiting our understanding of the representativeness of the sample. We also expect that some participant age data reflect data-entry errors (eg, children aged 1 year); although these data add noise, we have no way of identifying these children’s actual ages, and some youth younger than 10 years likely do access GenAI chatbots to some extent; we collapsed all users aged younger than 10 years for this reason but recognize inferences about this group may be incorrect or imprecise. Additionally, although we collect data from the child’s device on which parents install the Aura parental monitoring app, other users could ostensibly use the device (eg, a parent or sibling). Nonetheless, this approach preserved participant privacy and allowed the use of high-dimensional, deidentified, aggregated data, which reduced self-selection bias.

Aspects of the sampling framework via the Aura parental monitoring app may also introduce confounds when directly comparing age cohorts. Parental monitoring is common among US parents [40], yet caregivers may adopt these technologies for different reasons depending on their child’s age. For example, younger children who are already more prone to risk-taking or impressionability may elicit greater monitoring, whereas older youth may be more adept at evading detection (eg, by using peers’ devices).

The current data collection method also restricts analysis to users’ outgoing keystrokes—rather than considering the chatbots’ prompts or responses. GenAI systems may shape these conversations, such as by design features eliciting or nudging the user toward more or less risky conversation topics; more work is needed to understand the dynamics of GenAI interactions, chatbot design, and user agency across themes. Additionally, we only investigate the themes of behavior for users who both utilize GenAI and have the keyboard enabled. Participants could opt into or out of the overlay keyboard at any time, a procedural strategy that maximizes agency and privacy for the participants. Methodologically, this means that periods without recorded data may reflect either genuine nonuse of GenAI or active use that was not captured. Because these scenarios cannot be differentiated, true nonuse and missing data are conflated throughout, and findings should be interpreted accordingly. Engaging in more explicit or risky conversations likely also correlates with youth opting out of the Aura keyboard overlay, such that our data may systematically miss AI interactions that are more extreme, potentially problematic, or age-inappropriate. Thus, given this limitation—and even despite the explicit conversations we do observe in the data—our dataset likely underrepresents the amount of highly explicit or risky engagement youth have with AI. Ongoing prospective research with a diverse cohort addresses many of the limitations of the current project and will be available soon for analysis [41].

Our analysis is also limited to text-based (vs voice-based) conversations, limited solely to GenAI apps (vs a GenAI chatbot embedded within another app, such as Facebook or Snapchat), and limited to interactions via a mobile app (vs a browser). Finally, although our AI-based coding strategy was necessary to handle high-dimensional data at scale, it may have introduced coding errors. Specifically, LLMs can produce inconsistent outputs across repeated runs of similar inputs and are susceptible to hallucinations, whereby plausible but inaccurate codes may be generated. Our approach aimed to mitigate these issues by comparing the LLM coding with a human-coded sample and achieved good reliability. Human coders—given sufficient time and resources—might have mitigated issues related to LLM outputs.

The current study provides a critical preliminary step toward understanding how youth use AI chatbots and the potential risks of such use. Although accessing naturalistic user data presents inherent challenges in ensuring participant privacy, the public-interest value of understanding how children engage with AI necessitates innovative approaches to data collection. The current study exemplifies how academic-industry partnerships can responsibly leverage platform-derived data for scientific purposes aimed at safeguarding child well-being, rather than maximizing profit through user engagement. Protecting young users in the age of AI will require rapid-response research, greater transparency, independent oversight, and regulatory standards for AI companies that prioritize developmental well-being over engagement-driven design.

## Appendix

Multimedia Appendix 1 Detailed analysis phases, supplementary tables and figures, list of applications used, and the AI coding prompt.

## Abbreviations

GenAI: generative AI
LLM: large language model

## Data Availability

The aggregate quantitative data generated and analyzed during this study are available from the authors upon reasonable request and following completion of a data use agreement. The completed raw data are not publicly available to protect participant privacy.
