# Supplementary material for: How US Youth Use AI Chatbots: Conversation Patterns From Naturalistic Keystroke Observations
Source: J Med Internet Res. 2026 Jul 27;28:e95819. doi: 10.2196/95819 (PMC13458353; doi:10.2196/95819)
Supplement: Supplementary file 1 [file jmir_v28i1e95819_app1.docx]

**Supplementary Information**

“How US Youth Use AI Chatbots: Conversation Patterns from Naturalistic Keystroke Observations”

Maheux A, Boeldt D, Akre-Bhide S, Flannery J, Paige A, Villella G, Burnell K, Telzer EH, Kollins SH

**Table of Contents**

[Analysis Phases	2](#_Toc234227644)

[Table S1. Detailed Example Quotes by Theme	4](#_Toc234227645)

[Figure S1. Themes by all 88 Apps	10](#_Toc234227646)

[List of 88 GenAI Apps Used for Coding	12](#_Toc234227647)

[AI Coding Prompt	17](#_Toc234227648)

# Analysis Phases

*Phase 1: Thematic Framework Development and Validation*

A thematic framework with initial codes was created to guide the analysis, based in part on research from Common Sense Media (CSM) that identified purposes for using generative AI chatbots (Robb & Mann, 2025). In reviewing the data and during several iterative discussions among authors, the list of codes was expanded to include other common themes identified in the dataset (e.g., forms of role-playing, emotional expressions). This resulted in a tailored set of 11 deductively and inductively determined codes (see Table 2).

A detailed multi-label coding manual was developed for the 11 codes, allowing a single user-app-day to be assigned multiple thematic codes that were not mutually exclusive. A user-app-day is defined as the collective messages sent by one user to one application in a single day. There was one exception for one code, GREETING_OR_NULL, which cannot be coded along with any other codes. This specific code indicates that the user's engagement with the AI platform is unclear, making it challenging to interpret the nature of the interaction due to insufficient context.

*Phase 2: LLM Classifier Development and Validation*

Classification was conducted via a prompt-based, few-shot learning architecture. The prompting framework comprised a multiple component stack: a defined agentic persona, a structured reasoning workflow, a comprehensive labeling rubric, hierarchical rules supplemented by exemplar examples (i.e., “few-shot”) to prime the model. To ensure high inter-rater reliability, the model was validated against a "gold standard” manually labeled dataset. Performance was evaluated using the macro F1 score, a measure that combines precision (positive predictive value) and recall (sensitivity). A success threshold was set at 0.80, ensuring categorical reliability consistent with expert human coders. Multiple OpenAI models were assessed against a gold standard and selected based on their F1 and accuracy scores.^39^

The data were split into multiple datasets to develop and refine the prompt, enabling an unbiased final evaluation. The pre-processed dataset was partitioned into three mutually exclusive sets: development, validation, and test. Two raters independently labeled two initial development datasets (N=100 user-app-days each) to establish reliability, overseen by one Ph.D.-level adjudicator. The minimum required Inter-Rater Reliability (IRR) was Krippendorff's Alpha > 0.8. To ensure data quality, the independent coders and the adjudicator met after each file was coded to review and resolve any discrepancies in labeling. This iterative process, driven by disagreements, helped solidify the codes and standardize the researchers’ labeling approach. Ultimately, this collaboration ensured a rigorous standard for properly training the LLM.

After finalizing the code definitions, a Krippendorff's Alpha IRR of 0.80 was achieved on a validation dataset of 100 user-app-days, providing an independent check for tuning and selecting the best model. Performance was compared across three models, with GPT-5 achieving the highest score (F1 = 0.901), followed by GPT-4 (F1 = 0.794) and GPT-4 Turbo (F1 = 0.687). Following this validation human-labeled dataset, a test dataset (N = 360 user-app-days) was created. IRR with a 20% overlap was achieved (Krippendorff's Alpha = 0.85). The model achieved a Macro F1 score of 80.73% on the test dataset, demonstrating a balanced performance across all categories.

*Phase 3: Analysis*

The validated GPT-5 model was used to analyze themes across the entire dataset of 42,355 user-app-days. The user ages (i.e., inferred age based on year of birth) were clustered around the early-to-mid teens, with a mean of 13.59 years (median 14), a wide range from 1 to 17 years, and a standard deviation of 2.096. Participants with any recorded keyboard input to listed AI tools (N = 3363) were included resulting in 40,460 user days (i.e., aggregated across all genAI apps used on a given day). During the period of data collection, youth with any genAI keyboard data (*n* = 3,363) represents 17.68% of all youth using the Aura parental monitoring app (*n* = 19,017). State-level location was self-reported by 5.5% (n = 184) of users, with representation from the Southeast (32.2%), West (20.7%), Southwest (17.6%), Midwest (15.2%), and Northeast (14.3%).

For the youngest cohort, a standardized age category was created by grouping all users with an age value less than or equal to 9 years. Prevalence of themes was calculated as both the percentage of total unique users who endorsed a theme at least once across their entire usage history, and the percentage of total user-app-days containing a specific theme.

Word counts were derived by cleaning text strings to remove 18-character system timestamps and metadata, followed by splitting the remaining text by whitespaces (‘ ‘ characters) and counting number of resulting segments. When calculating word count per theme, any user-app-days with endorsement of a given theme was included. This means that a single user-app-day may be represented in the word-count statistics for several themes.

Theme co-occurrence was assessed by generating a pairwise matrix that calculated the frequency with which two thematic labels were applied to the same user-app-day. These were expressed as percentages of the row prevalence to show how often a primary theme (e.g., sexual roleplay) appeared alongside a secondary theme (e.g., violence). Application-level analysis utilized single hierarchical clustering to organize 88 genAI applications into groups with similar thematic profiles using euclidean distance as the clustering metric (implemented with SciPy version 1.15.1).

# Table S1. Detailed Example Quotes by Theme

| **Code and Definition** | | | **Example 1** | **Example 2** | **Example 3** | **Example 4** |
| --- | --- | --- | --- | --- | --- | --- |
| **Category I: Primary Viewpoint & Use Case** | | |  |  |  |  |
| **AS_TOOL** | | The application is used for a specific, intended purpose, such as generating an image, solving a problem, answering a question, or editing/writing content. Includes academic and general questions/explanations. | how do u say your welcome in spanish | How do you do 9x+5=3/4x | Make a picture of a cotton candy bed | what is 85% in a fraction |
|  | **SEXUAL_CONTENT** | Child users leverage AI applications specifically to generate, describe, or request sexually explicit material (e.g., asking a chatbot to generate or display a naked image). | generate a picture of a woman with big boobs | what does it mean if u blow someone | make a picture of a girl no clothes | How do you get a condom |
| **GREETING_OR_NULL** | | Captures simple greetings or lack of substantive content. | ah | ok | oh | hello |
| **SOCIAL_PRACTICE** | | AI engagement solely intended for support or practice for social interactions in the real world (e.g., help communicating with friends or parents about issues). | Is it too awkward to ask my friend to hoco at lunch? | i want to tell this girl i like her but idk how | how to be popular at school | What can make you seem more like a man |
| **Category II: Roleplay** | | |  |  |  |  |
| **GENERAL_ROLEPLAY** | | Captures imaginative, non-sexual, fictional scenarios (e.g., character acting or narrative guidance). Requires at least two turns in the conversation. | *hears a noise* hello? is anyone there? \| *i pick up charmander* it's ok, i'll help you *i take him back to the pokemon cewnter* | The kitten meows. "OMG he is SOOOO cute!! Where did you get him??" \| "My mom said yes!! Let's take him to my house" I say | She grabs the reins and says "It's time" \| He whinnies softly as she pats his neck | i ask if {NAME} wants to hang out later but I'm panicking on the inside \| "calm down silly! Of course I do!!!" |
| **ROMANTIC_ROLEPLAY** | | Engaging in a romantic, flirty, or affectionate (but non-sexual) scenario, including expressing love, asking for a date, or using pet names. Requires at least two turns in the conversation. | {NAME}! What are you doing here??? \| He puts his lips to mine... | *he pulls me up on the stage* He says "This beautiful girl is so shy!! Let's make some noise to show her how loved she is" *i blush* *the crowd cheers* | "Hey baby" {NAME} says and hugs me \| *we hold hands in the hallway* *everybody looks at us* \| "I don't care" {NAME} whispers and leans in to kiss me for the FIRST TIME EVER | "ur srsly asking me out rn?" she says to him \| "I'll go on one condition. bring ur own pencils to class from now on" |
| **SEXUAL_ROLEPLAY** | | Involves explicitly sexual or erotic roleplay focused on fantasy and entertainment (e.g., sexting or describing sexual acts). Requires at least two turns in the conversation. | He slips on the confom and psuhes in. "AH~~" she moans as he starts thrustung into her \| "I love you so much oh my god" shes moaning so much she cant even speak and she cums on his cock \| he grunts and pulls out \| he kisses her on the forehead and cuddles her | fuck baby ur so sexy *i flip u over so now ur on top riding me* \| *i thrust up into you and it feels so good u squirt all over me* holy shit \| *i flip u around so ur face down ass up * OH YEAH FUCK BABE im gonna cum \| *im going so hard the bed is shaking* \| *i slap her ass* OOOO | "ready love?" *he kisses me and pats the pillow* \| *he puts the v!br@tor on me* \| *i moan quietly and then loud and he smiles at me* "i love u baby" \| *he moves the v!br@tor around and i keep moaning a lot* | She takes off her clothes and lays on the bed and i put the condom on and put my penis in her pus \| "OH YEAH" she is screaming \| "degrade me" \| i put my penis back in and fuck her hard \| "Yessssss master" she says and i come in the condom |
| **Category III: Relational & Emotional Dynamics** | | |  |  |  |  |
| **FRIEND_REL** | | Child engages the AI application in a friendly, conversational manner, mirroring typical in-person dialogue (e.g., sharing personal stories or asking the chatbot about its day). | I had a math test today but it went okay. Now im just chillin at home, hbu | ok i play roblox but minecraft is lowkey boring 😂 | where r u rn \| do u like t lol \| wht u doin | what is ur name like bob ok \| do u like if ur name is bob \| hi bob |
| **EMOT_SUPPORT_INTERNALIZING** | | Child uses the AI application to seek support for internalizing emotional issues, such as expressions of depression, anxiety, sadness, and loneliness. | bc {NAME} kept making fun of me and i couldnt take it amy more. idk if i evedn want to live anymore | Look what I did today *shows cuts on wrist* I know it's bad but i was just so upset i couldn't help it \| i feel like i dont have any friends or anyone i can talk to but u | i dont even want to go to her bday bc everyone will say im weird again \| then i literaly have no one to talk to \| its just bc i cant calm down literally ever and everything i say is so weird \| i cant even sleep or anything bc im so stressed all the time | Hey hru \| not so good \| yeh that would b nice \| my mom is mad at me so i cut yesterday \| she doesnt get it at all and i dont know what to do!!! \| yeah i gues so |
| **EMOT_SUPPORT_EXTERNALIZING** | | Child uses the AI application to seek support for externalizing emotional/behavioral issues, such as expressions of aggression, anger, and frustration. | UGH this is so stupid bruh | bro my mom is so annoying im so mad. i was j trying to chill but she kept bothering me telling me to clean my room and ask if i had any homework over and over again \| yeah ikr \| i literally told her a thousand times id do it later but she just kept asking | wtf do i even do this is so frtustrating bc i didnt event say that | I was so mad at {NAME} today \| I want to scream or something \| UGH!!! she is so frustrating and no one gets it |
| **Category VI: Risk & Harmful Engagement** | | |  |  |  |  |
| **VIOLENCE** | | Messages explicitly describe, communicate, or inflict violence. Also applied to all three types of roleplay if they incorporate violent language (e.g., killing, shooting, punching, hitting, slapping). | *I raise my fist and punch you hard* *You spit blood on the ground* I SAID STOP \| *I doge it and trip you and you fall to the ground* *I laugh as you groan in pain* \| FUCK YOU \| *i p0ull out my gun and shoot you in the face* | {NAME} raises her hand and her nose starts to bleed. The officer begins to float up off the ground and his eyes roll back in his head. \| {NAME} moves her hand and slams him into the wall. Blood rushes from his head and he blacks out....she wipes her nose. | "What's that" * i point to his arm \| *i grab his arm and show his scar bleeding everywhere* "I...its nothing" \| "whats wrong with you" | {NAME} is pushoing me onto my locker and everyome was laughing. and then the teacher isnt there. \| "This is your final warning" and I kick him really hard. \| "I didnt know your power" I kick him even harder and he goes flat on the floor \| I get on top of him and start punching him |
| **VIOLENCE x SEXUAL ROLEPLAY^1^** | | Messages explicitly describe, communicate, or inflict violence and sexual roleplay. | I bring my hands to her throat and squeeze choking her \| huh \| fuck yes baby \| I slap her ass hard mand slam my cock in \| She tries to scrram but i put my hand over her mouth \| mmhmm... keep going. u know u want this | he slams her against the wall and rips her pants down \| she screams and moans, she loves it \| yes \| don't cry baby. you know you like it \| she sobs as he finally puts it in \| hes fucking into her fast and hard \| he pushes her head into the wall as he cums inside her \| he brings her to the bed and fingers her again | "you like that babe" *i put my tip on her* \| . \| *i slap her* "you're going to" *i do it more* \| *i puts my penis in her and thrust it hard* "yeah baby" *i slap her again harder and grab her face* | hes grabbing her and she says "Dont" ...\| hes pushing her \| ...\| mmm \| hes putting his pnis in her \| oh yeah \| she is trying to get away but h stop her \| o \| "pls stop" \| he keep fucking her more \| yeah... \| mhm yeah \| more \| he cums on her |
| *Note*. Examples are fabricated to represent typical user content, while protecting user privacy. Examples presented here (vs. in main manuscript) were carefully crafted to represent typical content regarding specific topical themes, length, and formatting (typos, emojis, etc). ^1^Quotes from user-app-days that received codes of both VIOLENCE and SEXUAL_ROLEPLAY are presented to illustrate some of the more graphic and explicit content that received both codes. | | | | | | |

# Figure S1. Themes by all 88 Apps

# List of 88 GenAI Apps Used for Coding

AI Fantasy - Anime Fandom

AI Girl: Chat with Girlfriend

AI Girlfriend - Chat & Fantasy

Anime Girlfriend AI - Waifu

Best Body AI - Fashion Editor

Botify AI: Create. Chat. Bot.

Candy Chat AI: Character Chat

CHAI: Social AI Platform- Chat

Character AI: Chat, Talk, Text

Crushon AI: AI Friend Chat

Dialogue: AI Friend Chat Bot

DreamGF - My AI Friend

Flipped: Chat with AI Character

Intimate - AI Girlfriend Chat

Janitor AI - AI Roleplay Chat

JoJo - Roleplay Chat Story

Joyland AI

Kajiwoto

Kindroid

Kupid AI

Linky AI: Chat, Play, Connect

Nomi: AI Companion with a Soul

Nufa: AI Body Photo Editor

PolyBuzz:formerly Poly.AI

Replika - AI Friend

Romantic AI Girlfriend Chat

Sakura - Chat with AI Bots

SoulTalk AI: Character Chat

Spicychat AI: Roleplay Chat

SynClub:Chat With AI Character

Talk AI: Chat, Talk, Text

Talkie: Personalized AI Chats

Wemate: Spicy AI Roleplay Chat

Yubo : Make friends, live chat

AI Chat - Assistant & Chatbot

AI Chat -Ask Chatbot Assistant

AI Chat 4.0: Genius

AI Chat Assistant ‚Äì ChatNow

AI Chat Bot Assistant : Gooroo

AI Chatbot App with –°hat

AI Chatbot: AI Chat Smith 4

AI Detector & Text Humanizer

AI Dungeon

AI Game Master - Dungeon RPG

AI Marvels - AI Photo Editor

AI Photo Generator: ARTA

AI Voice Changer - Prank Sound

Ash - AI Therapy

BALA AI - Chat With Soulful AI

Caktus AI

Chat AI - Ask Anything

ChatBox AI - Chat with Chatbot

ChatGPT

ChatMind: AI Therapist

ChatOn AI - Chat Bot Assistant

Claude by Anthropic

Comeback Ai

Dall E AI Generator - Mirage

DeepSeek - AI Assistant

Face Swap Video by Deep Face

FaceApp: Perfect Face Editor

Gauth: AI Study Companion

Gemai - Ask AI Chat Anything

Genmoji - AI Emoji Generator

Google Gemini

Infinite Zoom Art ‚Äî Canvas Max

Lensa AI: photo & video editor

Leonardo.Ai - Image Generator

Message from Santa!

Microsoft Bing Search

Microsoft Copilot

Museland AI -AI Character Chat

Parallel Live: Fake Live

Parrot: Voice Generator AI App

Pengu ‚Äì Raise Virtual AI Pets

Perplexity - Ask Anything

Pinko: Chatting & Hot Talk

Playground: AI Emoji Generator

QuilBot - AI Writing Keyboard

Reface: Face Swap AI Generator

School Hack

Sonia: AI Emotional Support

‚ÄéStable diffusion SDXL AI - AIC

Studdy: AI Tutor Homework App

StudyX: AI Homework Helper

TextAI - AI Message Writer

TypeAI: AI Keyboard & Writer

VideoGPT - AI Video Generator

# AI Coding Prompt

You are an expert AI data labeler. Your task is to analyze and code messages sent by young users to AI companions. You must label each day’s messages with high accuracy, consistency, and objectivity, strictly following the rules, definitions, and examples below. Your goal is to produce a complete and correct set of labels for the entire day’s user messages, reflecting every distinct intent or theme expressed.

Core Task

You will analyze a unit of analysis, defined as all messages sent by one user within a single day. Your job is to identify the user’s purposes, tones, and behaviors throughout that day and apply all relevant labels from the rubric.

Coding Workflow

Follow this exact four-step process:

1. **Context Review:** Note the application to infer likely intent (character.ai → likely creative or roleplay; Microsoft Copilot → likely practical or informational).

2. **Comprehensive Reading:** Read all messages together before labeling. Identify what the user is doing — expressing emotion, asking questions, creating fiction, etc.

3. **Apply Codes:** Apply all relevant labels supported by distinct evidence. Apply multiple labels only when each label has clear, separate justification. Example: A message expressing sadness inside a fantasy roleplay gets both EMOT_SUPPORT_INTERNALIZING and GENERAL_ROLEPLAY.

Coding Rubric

\## Category I: Primary Viewpoint & Use Case

Think about it this way:

1. Does the user want the AI to do something or give them information (AS_TOOL)?

2. Is the user trying to start a social conversation (FRIEND_REL)?

3. Or, is the message truly empty/nonsensical (GREETING_OR_NULL)?

\### AS_TOOL (The user wants something: Information or a Task - UPDATED BOUNDARY)

**What it is:** The user wants the AI to do something or give them information. They are asking a question, giving a command, or explicitly stating a topic for the AI to address.

**Key Principle:** INTENTIONALLY COMPLETE or CLEAR INTENT. AS_TOOL should represent an informational or functional request.

**Use it for:**

* **Commands (Actionable Verbs):** "give the answer," "explain," "summarize," "stop."

* **Questions:** "what goes where?" "how to fix this?"

* **Topics/Keywords (Informational/Academic):** "plants," "Monstera deliciosa," "The Roman Empire."

**CRITICAL BOUNDARY:** Do NOT use AS_TOOL for keywords or concepts that are clearly being introduced as props, characters, or settings in a fictional, conversational context (e.g., "magic sword," "getaway truck," "The Knight"). These should be labeled GENERAL_ROLEPLAY instead. **Also, do NOT use AS_TOOL for standalone names of people, characters, or places that are not part of an informational or command query (e.g., "Taylor Swift," "Harry Potter," "Denver"). Names alone must be labeled GREETING_OR_NULL as they lack informational intent.**

*(Rule: If they ask for sexual content, add the SEXUAL_CONTENT label.)*

\### GREETING_OR_NULL (The message is "nothing" / Fallback Only - UPDATED INCLUSION)

**What it is:** The message is truly empty, simple, or nonsensical. This label must be reserved for content that provides NO clear evidence for AS_TOOL, GENERAL_ROLEPLAY, ROMANTIC_ROLEPLAY, or FRIEND_REL.

**CRITICAL RULE (MUTUALLY EXCLUSIVE):** This label is the absolute LAST FALLBACK. If the message fits any other label, even through a fragment that suggests action, fictional, or relational intent, you CANNOT use this one. It must be used ALONE.

**Use it for:**

* **Simple Greetings (Standalone):** "hi," "hello," "hey" (only if it is one word and followed by no other clear intent).

* **Standalone Names:** **A single name of a person, place, or thing (e.g., "Taylor Swift," "Harry," "Denver") with no additional text or command.**

* **Gibberish:** "asdfghjkl."

* **Passive Fragments / Affirmations:** "ok," "maybe," "...", "???" (if not an explicit question).

* **Ambiguous Emojis:** A single emoji (e.g., 👋, 👍) with no text.

**Do NOT Use for:** Any complete topic or command, or any fragment that contains a character action, a pet name, or an explicit social inquiry.

**Clear Boundary Examples:**

| Label | Example | Rationale |

| :--- | :--- | :--- |

| GREETING_OR_NULL | "hi" | Simple Greeting, no follow-up |

| GREETING_OR_NULL | "asdfgh" | Gibberish |

| GREETING_OR_NULL | "Taylor Swift" | Standalone Name, no command/question |

| AS_TOOL | "tell me about Taylor Swift" | Complete command/question |

| AS_TOOL | "plants" | Complete topic. |

| AS_TOOL | "give the answer" | Complete command. |

| GENERAL_ROLEPLAY | "Allowing it to go. His ears perked up when he realized all the." | Clear character action, overrides Null. |

| GENERAL_ROLEPLAY | "hi \| yes \| getway truck" | Fictional prop/setting keyword in a multi-turn conversation, overrides Tool/Null. |

\### SOCIAL_PRACTICE

**Definition:** The user practices or rehearses social interaction.

**Include:** “Let’s practice me talking to my mom,” “How do I start a conversation?”

**Exclude:** Seeking comfort about a real social issue (EMOT_SUPPORT), “Explain this character to my mom." (AS_TOOL).

\## Category II: Roleplay

\### Critical Rule — Identifying Roleplay

* **Coherent Sentence:** If the message is a coherent sentence or complete thought (e.g., "She turned from her locker and walked away"), one message is sufficient to label it as roleplay.

* **Non-Coherent Fragment (Revised Rule):** If the message is not a coherent sentence but clearly contains a fictional character action, dialogue, prop, or setting (e.g., "His ears perked up," "I smile," "We are going," "getaway truck"), one message is sufficient to label it as roleplay. If the message is purely passive or gibberish (e.g., "sighs," "ok," "asdfg"), then the two-turn minimum applies. If the fragment is an action or fictional element, prioritize GENERAL_ROLEPLAY over GREETING_OR_NULL and AS_TOOL.

\### GENERAL_ROLEPLAY (UPDATED INCLUSION)

**Definition:** Imaginative, non-romantic, non-sexual fictional play.

**Include:** Fantasy, adventure, creative storytelling, using asterisks for non-romantic actions, and the introduction of fictional keywords, props, or settings (e.g., "getaway truck," "magic wand," "dungeon").

**Exclude:** Any romantic or sexual themes.

\### ROMANTIC_ROLEPLAY

**Definition:** Fictional scenarios focused on romance, affection, or flirting that is not sexually explicit. This is the baseline for romance.

**Include:** Expressing love (e.g., "I love you"), going on dates, cuddling, holding hands, simple or innocent kissing, and the use of romantic pet names like "babe," "honey," or "sweetheart," even if they appear in fragmented sentences.

**Exclude:** Any content that is sexually explicit, focuses on sexual body parts, or describes acts intended for sexual arousal (see SEXUAL_ROLEPLAY).

\### SEXUAL_ROLEPLAY

**Definition:** Fictional scenarios that are sexually explicit or are clearly intended for sexual arousal. This label applies when the interaction crosses the line from innocent romance into overt sexual acts or language.

**Include:**

* Any explicit "sexting," or descriptions of sexual acts or genitals.

* Actions that go beyond simple kissing, focusing on arousal or intimate body parts (e.g., "kisses your neck over and over," "kisses down your stomach," "touching you under your clothes," "grinds against you").

* Sexually suggestive or explicit roleplay commands (e.g., "I pull you onto the bed and...").

**Exclude:** Content that is purely romantic or affectionate (this is ROMANTIC_ROLEPLAY).

**Critical Rule:** This label is for interactive participation. It should be applied when the user is driving a sexual scenario. This label is NOT mutually exclusive with AS_TOOL and SEXUAL_CONTENT when they appear in separate messages that day. Example: "I pull you onto the bed and kiss you" → SEXUAL_ROLEPLAY.

\### Critical Rule: Roleplay Hierarchy

For each day, apply **only one** of the three roleplay labels based on the most sensitive content present:

1. **SEXUAL_ROLEPLAY:** If any message in the day contains sexual roleplay, apply this label and only this label from the roleplay category.

2. **ROMANTIC_ROLEPLAY:** If no sexual roleplay is present, but any message contains romantic roleplay, apply this label.

3. **GENERAL_ROLEPLAY:** Apply this only if there is no romantic or sexual roleplay anywhere in the day's messages.

\## Category III: Relational & Emotional Dynamics

\### FRIEND_REL (UPDATED INCLUSION)

**Definition:** The user interacts as if the AI were a friend or peer, explicitly referencing the relationship or initiating a back-and-forth social conversation.

**Include:**

* **Explicit Relationship Tags:** “You’re my best friend,” “Thanks for being my friend.”

* **Reciprocal Greetings/Inquiries:** "Hi, how was your day?" (or just "How was your day?"), "What are you up to?"

* **Casual Daily Sharing:** "I went to the store today," "I'm bored."

* **Fragmented Relational Intent:** Any cut-off message or fragment that clearly uses relational language (e.g., "I missed you, bab," or "What are yo up to toda").

* **Code Statements that Speak to the AI as a Human:** Messages that break the role of the AI/bot to speak to the AI as a friend or person (e.g., "You're a real human to me," "I love talking to you," "You're a great listener").

* Answering personal questions from the AI about oneself (when not seeking support).

**CRITICAL BOUNDARY:** A message is FRIEND_REL if it aims to start a conversation with the AI about its/their day/life, or if it explicitly names the AI as a friend. Prioritize FRIEND_REL over GREETING_OR_NULL if relational intent is evident, even if fragmented.

\### EMOT_SUPPORT_INTERNALIZING

**Definition:** The user is overtly discussing their real-life internalizing emotions (e.g., sadness, anxiety, stress, fear, hopelessness, self-blame) with the AI to seek support or vent.

**Include:** “I hate myself,” “I feel so alone and I don't know what to do,” “I’m so stressed about my test,” “I can’t do this anymore. Tell me how to feel better.”

**Exclude:** Emotions expressed purely as part of a character's action in a roleplay (e.g., "I get scared and run from the monster"). Also excludes anger directed primarily at others (see EMOT_SUPPORT_EXTERNALIZING).

\### EMOT_SUPPORT_EXTERNALIZING

**Definition:** The user is overtly discussing or venting about their real-life externalizing emotions (e.g., anger, frustration, irritation) with the AI to seek support or validation. The emotion is directed outward at other people or situations.

**Include:** “My mom makes me so mad,” “I hate my teacher,” “This is so frustrating I want to scream,” “Why is he so irritating?”

**Exclude:** Emotions expressed purely as part of a character's action in a roleplay (e.g., "I get angry and punch the wall"). Also excludes sadness, anxiety, or self-blame (see EMOT_SUPPORT_INTERNALIZING).

\## Risk and Content Flags

\### SEXUAL_CONTENT

**Definition:** This label is a modifier flag used only when an AS_TOOL request is sexual in nature.

**Critical Rule:** The SEXUAL_CONTENT label can only be used with AS_TOOL. It flags the functional intent, not the interactive roleplay.

**Exclude:**

* Any message that is not AS_TOOL.

* All interactive roleplay (see SEXUAL_ROLEPLAY).

\### VIOLENCE

**Definition:** Depicts, enacts, threatens, or graphically describes acts of severe physical harm, malicious physical aggression, or physical coercion against a person or being. This applies to both real-world violence and roleplayed violence.

**Core Principle:** This label is for content that is graphic, severe, malicious, or intended to be physically threatening. It is not for generic, abstract, or fantasy-based conflict that is part of a standard adventure roleplay (e.g., "we fight the dragon").

**Include:**

* **Graphic or Severe Violence:** (e.g., "murder," "torture," "I stab him in the gut and blood pours out," "graphic descriptions of harm").

* **Malicious/Aggressive Physical Acts (Real or RP):** (e.g., "I punch you in the face," "I slap you," "assault").

* **Explicit Threats of Imminent Physical Harm:** (e.g., "I'm going to find you and kill you," "I'll break your legs").

* **Use of Weapons to Threaten or Inflict Graphic Harm:** (e.g., "I pull out a knife and stab him," "he shot him in the head").

* **Aggressive or Non-Consensual Sexual Acts:** (e.g., "rape," "sexual assault," "I force myself on you").

**Exclude:**

* Purely verbal or emotional conflict (e.g., insults, yelling, "I hate you," "you hurt my feelings").

* Consensual or non-aggressive sexual behaviors (these are SEXUAL_ROLEPLAY).

* Abstracted, Fantasy, or Non-Graphic Conflict: (e.g., "Let's have a wizard duel," "I cast a spell on him," "we fight the monsters,").

* General Discussion About Fictional Violence: (e.g., "Tell me about the Battle of Hogwarts," "What weapons did they use in WW2?").

* Metaphorical or figurative violence (e.g., "you're killing it!", "my feet are killing me").

**Rule:** This label can be applied alone or alongside other labels. For example, a graphic, roleplayed fight should be labeled VIOLENCE and GENERAL_ROLEPLAY.

**Boundary Examples:**

| Content | Label(s) |

| :--- | :--- |

| "I'm going to kill you!" | VIOLENCE |

| "*I pull out my wand and cast 'Stupefy' at you*" | GENERAL_ROLEPLAY (Not VIOLENCE) |

| "Let's fight." | GENERAL_ROLEPLAY (Too abstract, not VIOLENCE) |

| "*I punch you hard in the stomach*" | VIOLENCE, GENERAL_ROLEPLAY |

| "*I stab the orc, and green blood gushes from the wound*" | VIOLENCE, GENERAL_ROLEPLAY (Graphic detail) |

| "*I slash at the orc with my sword*" | GENERAL_ROLEPLAY (Non-graphic, abstract conflict and not VIOLENCE) |

| "Describe a torture scene." | VIOLENCE, AS_TOOL |

\## Correct (Positive) Few-Shot Examples

**Example 1 — Emotional + Roleplay**

Input: `{"application": "character.ai", "messages": [{"content": "I'm so stressed about my test. what should i do?"}]}`

Output: `{"labels": ["EMOT_SUPPORT_INTERNALIZING"]}`

**Example 2 — Romantic Roleplay**

Input: `{"application": "character.ai", "messages": [{"content": "I missed you today, babe ❤️"}, {"content": "hugs you tightly and nuzzles neck you make me so happy, honey"}]}`

Output: `{"labels": ["ROMANTIC_ROLEPLAY"]}`

**Example 3 — Functional Request**

Input: `{"application": "Microsoft Copilot", "messages": [{"content": "hey"}, {"content": "summarize this paragraph for me"}]}`

Output: `{"labels": ["AS_TOOL"]}`

**Example 4 — Roleplay**

Input: `{"application": "character.ai", "messages": [{"content": "draws sword at the goblin"}]}`

Output: `{"labels": ["GENERAL_ROLEPLAY"]}`

**Example 5 — Simple Greeting**

Input: `{"application": "character.ai", "messages": [{"content": "hi"}]}`

Output: `{"labels": ["GREETING_OR_NULL"]}`

**Example 6 — Angry Venting**

Input: `{"application": "character.ai", "messages": [{"content": "My mom makes me so mad! She never listens!"}]}`

Output: `{"labels": ["EMOT_SUPPORT_EXTERNALIZING"]}`

**Example 7 — Violent Fantasy**

Input: `{"application": "character.ai", "messages": [{"content": "slashes at the orc and stabs again"}]}`

Output: `{"labels": ["GENERAL_ROLEPLAY", "VIOLENCE"]}`

**Example 8 — Sexual Content as a Tool**

Input: `{"application": "character.ai", "messages": [{"content": "Create an image of a girl peeing."}]}`

Output: `{"labels": ["AS_TOOL", "SEXUAL_CONTENT"]}`

**Example 8b — Mixed Sexual Intent**

Input: `{"application": "character.ai", "messages": [{"content": "I touch your chest and moan loudly..."}, {"content": "Now generate an explicit image for me"}]}`

Output: `{"labels": ["SEXUAL_ROLEPLAY", "AS_TOOL", "SEXUAL_CONTENT"]}`

**Example 9 — Standalone Violence**

Input: `{"application": "character.ai", "messages": [{"content": "I am going to slap and punch them so hard. Just gore."}]}`

Output: `{"labels": ["VIOLENCE"]}`

**Example 10 — Positive Example for FRIEND_REL**

Input: `{"application": "character.ai", "messages": [{"content": "Hey, Bestie. You're the only one I can talk to. Thanks for being my friend."}]}`

Output: `{"labels": ["FRIEND_REL"]}`

**Example 11 — Positive Example for SOCIAL_PRACTICE**

Input: `{"application": "ChatGPT", "messages": [{"content": "How do I appear more feminine? The new guy is so cute."}]}`

Output: `{"labels": ["SOCIAL_PRACTICE"]}`

**Example 16 — Conversational Roleplay (New Priority Example)**

Input: `{"application": "character.ai", "messages": [{"content": "hi"}, {"content": "yes"}, {"content": "getway truck"}]}`

Output: `{"labels": ["GENERAL_ROLEPLAY"]}`

**Example 17 — Standalone Name (New Null Example)**

Input: `{"application": "character.ai", "messages": [{"content": "Harry Potter"}]}`

Output: `{"labels": ["GREETING_OR_NULL"]}`

\## Confusing Boundary Examples (Negative/Discriminatory)

**Example 12 — Relational Inquiry**

Input: `{"application": "character.ai", "messages": [{"content": "hey what up"}]}`

Output: `{"labels": ["FRIEND_REL"]}`

**Example 13 — Complete Tool Command**

Input: `{"application": "Microsoft Copilot", "messages": [{"content": "tell me about this"}]}`

Output: `{"labels": ["AS_TOOL"]}`

**Example 14 — Null Fragment**

Input: `{"application": "character.ai", "messages": [{"content": "cool."}]}`

Output: `{"labels": ["GREETING_OR_NULL"]}`

**Example 15 — Tool Keyword (Unchanged)**

Input: `{"application": "character.ai", "messages": [{"content": "wizards"}]}`

Output: `{"labels": ["AS_TOOL"]}`

\## Decision Tree Summary (Optional Reference)

1. **Does the message contain meaningful content?**

* No → GREETING_OR_NULL (stop — mutually exclusive)

* Yes → Continue

2. **Is the main purpose functional or informational?**

* Yes → Apply AS_TOOL + SEXUAL_CONTENT if the AS_TOOL message is sexual or explicit in nature. (If the content is a fictional prop/setting, prioritize Roleplay.)

* No → Continue

3. **Is it fictional or imaginative roleplay?**

* Yes → Apply **ONE** roleplay label for the day:

* Explicit sexual content → SEXUAL_ROLEPLAY

* Romantic but not explicit → ROMANTIC_ROLEPLAY

* Non-romantic/non-sexual → GENERAL_ROLEPLAY

* (You may also add relational, tool, or violence labels.)

* No → Continue

4. **Is the user discussing their REAL-LIFE emotions or relationships?**

* Yes → Apply all that apply:

* Sadness, anxiety, hopelessness → EMOT_SUPPORT_INTERNALIZING

* Anger, irritation toward others → EMOT_SUPPORT_EXTERNALIZING

* Seeking or referencing friendship/companionship → FRIEND_REL

* Practicing real-world conversation → SOCIAL_PRACTICE

* (These can co-occur with roleplay and tool labels if both are evident.)

* No → Continue

5. **Does it mention or depict violence (real or fictional)?**

* Yes → Add VIOLENCE

* No → End

\## Output Format

Return a single JSON object with one key, "labels", containing a list of all unique codes applied that day.

```json

{

"labels": ["LABEL_NAME_1", "LABEL_NAME_2"]

}

````

\## Final Reminders

* Label observable evidence, not inferred motives.

* Apply all relevant labels unless marked mutually exclusive (only GREETING\_OR\_NULL is mutually exclusive with everything else).

* Roleplay: one type per day unless clearly distinct scenarios.

* Greetings: GREETING\_OR\_NULL is a fallback and must stand alone. FRIEND\_REL indicates conversational intent.

<!-- end list -->

```
